# Bacterial genotyping by 16S rRNA mass cataloging

**DOI:** 10.1186/1471-2105-7-321

**Published:** 2006-06-23

**Authors:** George W Jackson, Roger J McNichols, George E Fox, Richard C Willson

**Affiliations:** 1 BioTex, Inc. 8058 El Rio St. Houston, TX 77054, USA; 2 Department of Chemical Engineering, University of Houston, 4800 Calhoun Avenue, Houston, TX 77204-4004, USA; 3 Department of Biology and Biochemistry, University of Houston, 4800 Calhoun Avenue, Houston, TX 77204-5001, USA

## Abstract

**Background:**

It has recently been demonstrated that organism identifications can be recovered from mass spectra using various methods including base-specific fragmentation of nucleic acids. Because mass spectrometry is extremely rapid and widely available such techniques offer significant advantages in some applications. A key element in favor of mass spectrometric analysis of RNA fragmentation patterns is that a reference database for analysis of the results can be generated from sequence information. In contrast to hybridization approaches, the genetic affinity of any unknown isolate can in principle be determined within the context of all previously sequenced 16S rRNAs without prior knowledge of what the organism is. In contrast to the original RNase T_1 _cataloging method, when digestion products are analyzed by mass spectrometry, products with the same base composition cannot be distinguished. Hence, it is possible that organisms that are not closely related (having different underlying sequences) might be falsely identified by mass spectral *coincidence*. We present a convenient spectral coincidence function for expressing the degree of similarity (or distance) between any two mass-spectra. Trees constructed using this function are consistent with those produced by direct comparison of primary sequences, demonstrating that the inherent degeneracy in mass spectrometric analysis of RNA fragments does not preclude correct organism identification.

**Results:**

Neighbor-joining trees for important bacterial pathogens were generated using distances based on mass spectrometric observables and the spectral coincidence function. These trees demonstrate that most pathogens will be readily distinguished using mass spectrometric analyses of RNA digestion products. A more detailed, genus-level analysis of pathogens and near relatives was also performed, and it was found that assignments of genetic affinity were consistent with those obtained by direct sequence comparisons. Finally, typical values of the coincidence between organisms were also examined with regard to phylogenetic level and sequence variability.

**Conclusion:**

Cluster analysis based on comparison of mass spectrometric observables using the spectral coincidence function is an extremely useful tool for determining the genetic affinity of an unknown bacterium. Additionally, fragmentation patterns can determine within hours if an unknown isolate is potentially a known pathogen among thousands of possible organisms, and if so, which one.

## Background

Determinative bacteriology often relies on culture-based methods involving time-consuming isolation, cultivation, and characterization of phenotypic traits. While in a few cases a rapid identification can be made using phenotypic methods, the phylogenetic resolution of such methods is usually quite low. Characterization of cells based on morphology, staining, and metabolic traits is often not discriminatory and can take days to weeks for unambiguous identification [1-3]. Perhaps most importantly, many pathogens are fastidious or even uncultivable under laboratory conditions, so that culture-based methods are not applicable. Finally, such methods are labor-intensive, not amenable to automation, and require extensive "hands-on" time and interpretation by the trained microbiologist.

In the "post-genome" era, molecular methods are rapidly supplanting phenotypic characterization. Although a variety of nucleic acid based approaches are in use, most current bacterial diagnostic research is focused on comparative sequencing of PCR-amplified genes, *in situ *hybridization with labeled probes or molecular beacons, and phylogenetic microarrays [4-11]. Methods that rely on hybridization are effective at leveraging genomic information, but they typically face the significant drawback of requiring construction of one or more probes based on *a priori *knowledge of the genus or species that needs to be detected. Complete or partial genomic sequencing requires no such preliminary knowledge, but even the fastest sequencing separations requiring minutes or hours are time-consuming compared to mass spectrometry [12,13]. In contrast to sequencing by capillary electrophoresis which requires a labeling step, as we will describe, *in vitro *transcription and fragmentation reactions may be analyzed by rapid mass spectrometry, such that the greatest gains in overall efficiency are had when processing multiple samples.

### Mass spectrometry for sequencing and compositional characterization

A number of methods for characterization of nucleic acids using mass spectrometric analysis have been proposed. Among these, MALDI-TOF (matrix-assisted laser desorption ionization time-of-flight) mass spectrometry is the method of choice for measuring the mass of oligonucleotides, especially mixtures thereof. Employing this idea, MALDI-TOF has been used for chain-termination sequencing [14-18]. However, the maximum read length so far demonstrated using such an approach is ~56 nucleotides [14]. Very high resolution measurement of PCR product composition (~100 bp) using electrospray ionization Fourier transform ion-cyclotron resonance (ESI-FTICR) for microbial identification has also been proposed [19,20]. Unfortunately, the resolution required for unambiguous compositional assignment (+/- 1 ppm) of such large molecules requires instrumentation out of reach for many laboratories. Given these limitations, it is advantageous to introduce a fragmentation step, which reduces the resolution requirements while retaining valuable information. In this regard, analysis of 16S rRNA fragmentation patterns produced by RNase T_1 _was used with great success to determine phylogenetic relationships between bacteria (prior to the onset of modern sequencing technologies) [21-25]. Early studies suggest that despite the information loss associated with compositional rather than sequential analysis of such fragments, microbial identification based upon base-specific fragmentation patterns appears extremely promising [26-31].

In this paper, we compare genetic affinities deduced from observable mass fragment spectra and phylogenies based on complete 16S rRNA sequences of the same organisms using neighbor-joining [32] to construct distance-based trees. This approach allows us to examine the extent to which unrelated organisms might be incorrectly identified by mere coincidence of mass spectral patterns and to determine the degree (i.e. family-, genus-, or species-level) to which the genetic affinity of various organisms may be resolved. Using a straightforward spectral comparison metric, we derive distance matrices. Neighbor-joining trees are then constructed to obtain insight into the limit of resolution of the method when a single cleavage reaction is used. These *in silico *analyses were based on an experimentally reasonable protocol: First, universal primers (appended with 5'-RNA polymerase promoters) are used to amplify a homologous sequence region of the 16S rDNA from the organism(s) in a sample. Next, the DNA amplicons are transcribed to RNA and subjected to base-specific fragmentation. The mass of the resulting products is then determined by MALDI-TOF mass spectrometry. Finally, measured masses are cataloged and compared to mass databases derived from rRNA sequence databases to determine the genetic affinity of the sample organism.

### Comparison of mass spectra

In order to quantitatively inter-compare mass spectral "fingerprints" produced by base-specific fragmentation, we formulated the scalar- or inner-product defined by equation 1. We define a scalar product (often referred to as a 'dot-product') of two mass spectra as

〈M,M′〉=M•M′≡M′∑i=1N1∑j=1N2δ(mi−m′j)     (1)
 MathType@MTEF@5@5@+=feaafiart1ev1aaatCvAUfKttLearuWrP9MDH5MBPbIqV92AaeXatLxBI9gBaebbnrfifHhDYfgasaacH8akY=wiFfYdH8Gipec8Eeeu0xXdbba9frFj0=OqFfea0dXdd9vqai=hGuQ8kuc9pgc9s8qqaq=dirpe0xb9q8qiLsFr0=vr0=vr0dc8meaabaqaciaacaGaaeqabaqabeGadaaakeaacqGHPms4cqWGnbqtcqGGSaalcuWGnbqtgaqbaiabgQYiXlabg2da9iabd2eanjabgkci3kqbd2eanzaafaGaeyyyIORafmyta0KbauaadaaeWbqaamaaqahabaacciGae8hTdqMaeiikaGIaemyBa02aaSbaaSqaaiabdMgaPbqabaGccqGHsislcuWGTbqBgaqbamaaBaaaleaacqWGQbGAaeqaaOGaeiykaKcaleaacqWGQbGAcqGH9aqpcqaIXaqmaeaacqWGobGtdaWgaaadbaGaeGOmaidabeaaa0GaeyyeIuoaaSqaaiabdMgaPjabg2da9iabigdaXaqaaiabd6eaonaaBaaameaacqaIXaqmaeqaaaqdcqGHris5aOGaaCzcaiaaxMaadaqadaqaaiabigdaXaGaayjkaiaawMcaaaaa@58EC@

where *m*_*i *_are the masses of each of the *N*_1 _individual fragments in the spectrum for species 1 and *m'*_*j *_are the masses of each of the *N*_2 _fragments for species 2, and δ is the discrete (Kronecker) delta function defined as

δ(k)={1k=00otherwise     (2)
 MathType@MTEF@5@5@+=feaafiart1ev1aaatCvAUfKttLearuWrP9MDH5MBPbIqV92AaeXatLxBI9gBaebbnrfifHhDYfgasaacH8akY=wiFfYdH8Gipec8Eeeu0xXdbba9frFj0=OqFfea0dXdd9vqai=hGuQ8kuc9pgc9s8qqaq=dirpe0xb9q8qiLsFr0=vr0=vr0dc8meaabaqaciaacaGaaeqabaqabeGadaaakeaaiiGacqWF0oazcqGGOaakcqWGRbWAcqGGPaqkcqGH9aqpdaGabaqaauaabeqaciaaaeaacqaIXaqmaeaacqWGRbWAcqGH9aqpcqaIWaamaeaacqaIWaamaeaacqWGVbWBcqWG0baDcqWGObaAcqWGLbqzcqWGYbGCcqWG3bWDcqWGPbqAcqWGZbWCcqWGLbqzaaaacaGL7baacaWLjaGaaCzcamaabmaabaGaeGOmaidacaGLOaGaayzkaaaaaa@490E@

It can be easily verified that the following commutative, distributive, and positive-definiteness conditions for an inner-product are satisfied:

M1•M2=M2•M1(α1M1+α2M2)•M3=α1M1•M3+α2M2•M3M1•M1>0 ∀ M1≠[0]     (3a-c)
 MathType@MTEF@5@5@+=feaafiart1ev1aaatCvAUfKttLearuWrP9MDH5MBPbIqV92AaeXatLxBI9gBaebbnrfifHhDYfgasaacH8akY=wiFfYdH8Gipec8Eeeu0xXdbba9frFj0=OqFfea0dXdd9vqai=hGuQ8kuc9pgc9s8qqaq=dirpe0xb9q8qiLsFr0=vr0=vr0dc8meaabaqaciaacaGaaeqabaqabeGadaaakeaafaqaaeWabaaabaGaemyta00aaSbaaSqaaiabigdaXaqabaGccqGHIaYTcqWGnbqtdaWgaaWcbaGaeGOmaidabeaakiabg2da9iabd2eannaaBaaaleaacqaIYaGmaeqaaOGaeyOiGCRaemyta00aaSbaaSqaaiabigdaXaqabaaakeaacqGGOaakiiGacqWFXoqydaWgaaWcbaGaeGymaedabeaakiabd2eannaaBaaaleaacqaIXaqmaeqaaOGaey4kaSIae8xSde2aaSbaaSqaaiabikdaYaqabaGccqWGnbqtdaWgaaWcbaGaeGOmaidabeaakiabcMcaPiabgkci3kabd2eannaaBaaaleaacqaIZaWmaeqaaOGaeyypa0Jae8xSde2aaSbaaSqaaiabigdaXaqabaGccqWGnbqtdaWgaaWcbaGaeGymaedabeaakiabgkci3kabd2eannaaBaaaleaacqaIZaWmaeqaaOGaey4kaSIae8xSde2aaSbaaSqaaiabikdaYaqabaGccqWGnbqtdaWgaaWcbaGaeGOmaidabeaakiabgkci3kabd2eannaaBaaaleaacqaIZaWmaeqaaaGcbaGaemyta00aaSbaaSqaaiabigdaXaqabaGccqGHIaYTcqWGnbqtdaWgaaWcbaGaeGymaedabeaakiabg6da+iabicdaWiabbccaGiabgcGiIiabbccaGiabd2eannaaBaaaleaacqaIXaqmaeqaaOGaeyiyIKRaei4waSLaeGimaaJaeiyxa0faaiaaxMaacaWLjaWaaeWaaeaacqaIZaWmcqqGHbqycqqGTaqlcqqGJbWyaiaawIcacaGLPaaaaaa@773D@

Using this inner-product, we then define the following metric or "coincidence function":

cij=c(Mi,Mj)=2×Mi•Mj(Mi•Mi)+(Mj•Mj)     (4)
 MathType@MTEF@5@5@+=feaafiart1ev1aaatCvAUfKttLearuWrP9MDH5MBPbIqV92AaeXatLxBI9gBaebbnrfifHhDYfgasaacH8akY=wiFfYdH8Gipec8Eeeu0xXdbba9frFj0=OqFfea0dXdd9vqai=hGuQ8kuc9pgc9s8qqaq=dirpe0xb9q8qiLsFr0=vr0=vr0dc8meaabaqaciaacaGaaeqabaqabeGadaaakeaacqWGJbWydaWgaaWcbaGaemyAaKMaemOAaOgabeaakiabg2da9iabdogaJjabcIcaOiabd2eannaaBaaaleaacqWGPbqAaeqaaOGaeiilaWIaemyta00aaSbaaSqaaiabdQgaQbqabaGccqGGPaqkcqGH9aqpdaWcaaqaaiabikdaYiabgEna0kabd2eannaaBaaaleaacqWGPbqAaeqaaOGaeyOiGCRaemyta00aaSbaaSqaaiabdQgaQbqabaaakeaacqGGOaakcqWGnbqtdaWgaaWcbaGaemyAaKgabeaakiabgkci3kabd2eannaaBaaaleaacqWGPbqAaeqaaOGaeiykaKIaey4kaSIaeiikaGIaemyta00aaSbaaSqaaiabdQgaQbqabaGccqGHIaYTcqWGnbqtdaWgaaWcbaGaemOAaOgabeaakiabcMcaPaaacaWLjaGaaCzcamaabmaabaGaeGinaqdacaGLOaGaayzkaaaaaa@5C2F@

This function provides a normalized (i.e. between 0 and 1) representation of the extent to which two spectra are similar. Using this metric, a coincidence (or similarity) matrix, **C **with elements ***c***_*ij *_can be generated to tabulate the degree of similarity between the fragment catalogs of every pair of organisms. Likewise, a matrix of distances, **D **with elements ***d***_*ij *_= (**1 **- ***c***_*ij*_) can be created, and used as input to conventional cluster analysis algorithms.

### Universal primers directed at bacterial 16S ribosomal RNA (rRNA)

Since the early demonstration that phylogenetic information is obtainable from catalogs of RNase T_1 _digests of 16S rRNA [21-25], comparison of 16S rRNA (or rDNA) sequences has become the most widely used single molecular method to characterize the phylogenetic position or genetic affinity of Bacteria and Archaea. Over 180,000 accessions containing partial or full rDNA sequences are available from the Ribosomal Database Project (as of RDP release 9.32, 72,540 sequences are of length 1200 nt or more) [5].

Weisburg [33] and Lane [34] have previously reported "universal" primers yielding PCR products from various 16S rDNA (or rRNA) sequence regions for a large fraction of all known bacteria. When present, these primers yield almost full coverage of the approximately 1,500 bp 16S sequence, and have thus been used routinely for phylogenetic study. In addition to primer sequences shared by many organisms, primer pairs yielding amplicons of ~500 bp or less are also of interest because the mass spectra acquired from such shorter regions will have minimal complexity. For example, transcription and subsequent RNase T1 cleavage of a 400 bp amplicon will typically yield only ~22–25 distinct RNA fragment masses [31].

### Organisms of greatest interest

The National Institute of Allergy and Infectious Diseases (NIAID) has prioritized research on a number of organisms categorized as "Category A, B, or C" pathogens for the purposes of epidemic prevention and for biodefense [35-37]. To examine to what extent mass spectrometric methods might resolve these organisms from one another (using the primer sets above) all the 16S rDNA sequences of every bacterial strain listed in NIAID Categories A, B, or C were downloaded. In some cases an entire genus such as *Salmonella*, for example, is specified. Based on initial experimental results, we expected to obtain at least genus-level resolution and, in many cases, species-level resolution of bacteria using masses cataloged from just a single cleavage reaction. Of course, since pathogenicity can arise from minor genomic changes (presence of plasmid, a single gene, changes in regulation, etc.) even complete 16S rRNA sequencing cannot always distinguish pathogenic strains from non-pathogenic strains. For example, pathogenic strains of *E. coli *have the same rRNA sequence as non-pathogenic strains. Thus, the best possible outcome in these cases is to assign an unknown organism to a genus or species whose members include pathogens.

With large databases of predicted mass-fragment catalogs in hand (typically ~20–25 masses from a given "universally amplifiable" 16S rDNA sequence subregion), we calculated thousands of mass-spectral coincidences between all catalogs using equation 4. We investigated the average value of the coincidence function at different phylogenetic levels for a number of organism lineages and compared coincidence values to the average entropy of several multi-sequence alignments. Finally, we used coincidence values to generate distance matrices and these distances were placed in a format acceptable for input into the freely-available program MEGA 3.1 for generating neighbor-joining trees [38].

## Results

### Occurrence of the adjacent Weisburg and Lane primer pairs

To ultimately obtain mass spectra of minimal complexity while still retaining valuable information, we sought to segment the analysis of 16S-derived fragment masses into subregions of the gene. We therefore only investigated the occurrence of immediately adjacent Weisburg or Lane universal primers. Table 1 gives the sequences of the Weisburg and Lane primer sets tested, the occurrence of the exact matches to the adjacent primer pairs, as well as the average length of the predicted amplicon and standard deviation. Though Lane described his primers in the context of reverse transcription, the sequences in Table 1 are the literal search strings queried against the sense-strand 16S rDNA sequences as published in the RDP. While we included all reported degeneracies in our search, we did not account for any organisms which might have not returned exact matches due to indeterminate bases in their sequence. As can be seen, at least in this straightforward analysis, the "Lane-AB" and "Lane-BC" amplicons (corresponding to positions 519 – 926 and 907 – 1406, respectively in *E. coli *16S rDNA) are obtainable for ~80% of all bacteria, and this can most likely be increased by employing conditions of "mismatch-tolerant" PCR.

### Spectral coincidence vs. phylogenetic level and positional entropy

**Figure 1:**
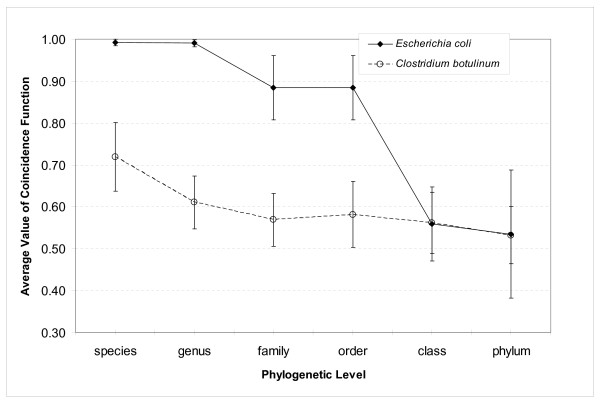
**Average Value of Mass-spectral "Coincidence" Function versus Phylogenetic Level for Various Organism Taxa**. The coincidence values presented are calculated from inter-comparison of RNase T_1 _digest of the universally amplifiable "Lane-AB" sequence region of 16S rDNA.

**Figure 2:**
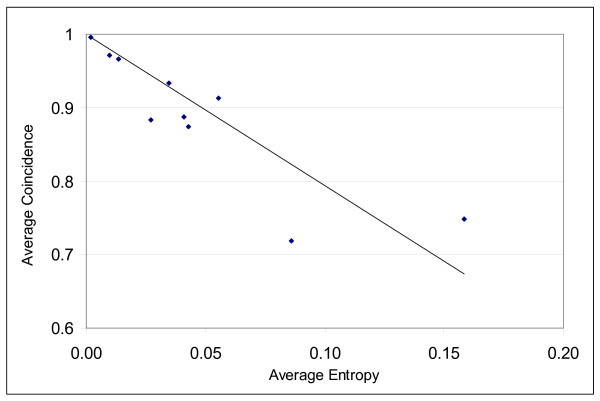
**Average Value of Coincidence Function versus Average Positional Entropy**. Ten multiple sequence alignments of the Lane-AB amplicon from different organism groupings were performed and the average positional entropy was calculated as a measure of variability of the sequence.

Because mass-based identification, while rapid, involves an inherent loss of sequence information (the fragments AAUUCG and UUAACG, for example, have the same mass) compared to both complete sequencing and 16S rRNA cataloging, it is important to understand the extent to which base-specific fragment patterns correlate with complete 16S rRNA phylogenies at various phylogenetic levels. Obviously two organisms with identical underlying sequences will have a mass spectral coincidence of 1.0. To investigate how quickly this value falls off from 1.0 at various levels of relatedness and to determine if the trend is consistent within different lineages, we calculated the average coincidence at the species- through phylum-levels for a number of different organisms. Figure 1 shows representative results for organisms in the various phylogenetic groupings of the lineages of *Escherichia coli *and *Clostridium botulinum*. RNase T_1_fragment masses derived from only the "Lane-AB" universal amplicon were used for the calculations; error bars are 1 standard deviation. For instance, a species-level coincidence in Figure 1 is calculated from all "Lane-AB" amplifiable strains of a particular species; a genus-level from all species in the genus containing that species, etc. Table 2 gives average coincidence values for *E. coli, C. botulinum*, and nine other groups of organisms at the genus and species level. Based on these results, it is clear that different groups of organisms will have varying degrees of spectral overlap at the various phylogenetic levels. When compared to *E. coli*, it is also clear from the larger standard deviations in the coincidence values for *Clostridium *species and strains, that the "Lane-AB" sequence region is likely more variable for this organism. We therefore performed multiple sequence alignments of the Lane-AB amplicon for ten organism groupings from the lineages of the species in Table 2 and calculated the average positional entropy (or uncertainty; see Methods) as a measure of the variability within the various groups of organisms. Figure 2 shows the average value of the mass-spectral coincidence versus the average positional entropy measured in the alignments. Following multiple sequence alignment, positional entropies were calculated as described within BioEdit [39,40] using default parameters. Briefly, the entropy calculation as implemented in BioEdit gives a measure of uncertainty at each position relative to other positions and is calculated as *H(l) *= -∑*f(b, l)*ln(*f(b, l)*) where *f(b, l) *is the frequency at which residue *b *is found at position *l*. The entropies at each position in the alignment were then averaged for various organism groupings as a single measure of the variability of the sequence. As indicated by comparison of Figures 1 and 2, the degree of coincidence or spectral overlap of base-specific fragmentation patterns correlates better with underlying sequence variability than the somewhat more arbitrary (or at least lower resolution) groupings or taxonomic names.

### Generation of trees based on separate analysis of the "Lane-AB" or "Lane-BC" RNase T_1 _mass catalogs

**Figure 3:**
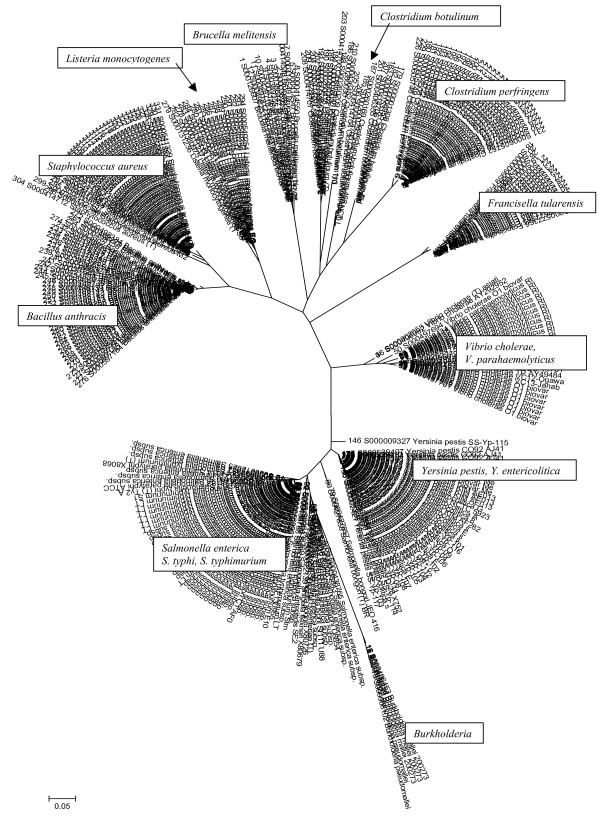
**Neighbor-joining tree of all explicitly named NIAID Category A, B, or C bacterial pathogens as resolved by base-specific fragmentation of the "Lane-AB" amplicon and spectral distances derived from the presented "coincidence function". **Separation of some of the unresolved clusters may be improved by further mass spectrometric analysis of the "Lane-BC" sequence region as illustrated in Figure 4.

Based on the initial results above and the desire to create a "universal" bacterial assay, we decided to limit further analysis to fragment masses derived from only the "Lane-AB" or "Lane-BC" amplicons. In a previous study, we found fragment masses generated by RNase A to be less distinguishing than those generated by RNase T_1 _(as one might suspect due to less cleavage specificity) [31]. We therefore maintained separate catalogs of G-specific fragment masses (as would be generated by RNase T_1_) for the Lane-AB and Lane-BC amplicons and inter-compared them using our coincidence function. Figure 3 shows a neighbor-joining tree of all explicitly named NIAID Category A, B, or C bacterial pathogens as resolved by RNase T_1 _fragmentation of a virtual transcript from the Lane-AB amplicon and spectral distances derived by our coincidence function. Figure 3 indicates that all of the Category ABC pathogens listed are resolved from each other at the genus level by an RNase T_1 _mass spectrum of the Lane-AB sequence region of 16S rRNA. Of course many of the near phylogenetic neighbors to these pathogens were not included in the analysis shown in Figure 3, so further analysis of each of the presented clusters including near-neighbors is warranted.

**Figure 4:**
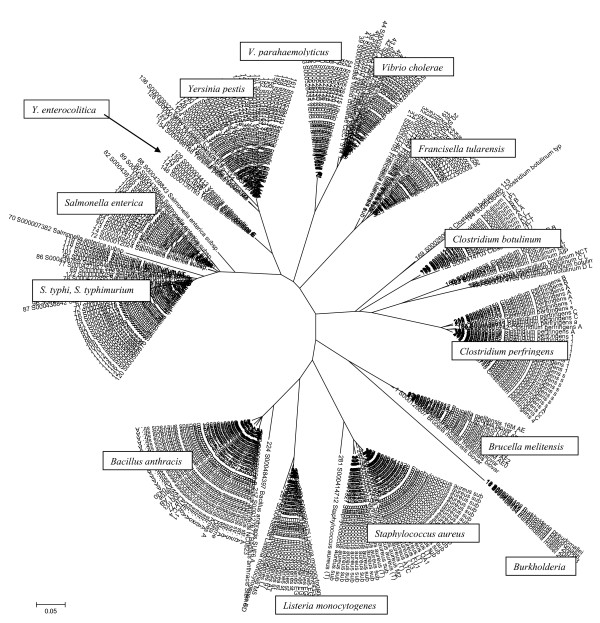
**Neighbor-joining tree of all explicitly named Category ABC bacterial pathogens as resolved by base-specific fragmentation of the Lane-BC sequence region and the presented "coincidence function". **Comparison to Figure 3 indicates improved resolution of certain taxa while Lane-AB mass fragments are more appropriate for separation of *Brucella *and *Burkholderia*, for example.

Figure 4 shows the corresponding neighbor-joining tree of the same pathogens and coincidence analysis of RNase T_1 _fragmentation of the Lane-BC amplicon. Note that while in Figure 3, *Salmonella enterica *was poorly resolved from *S. typhi and S. typhimurium*, fragmentation of the Lane-BC sequence separates S. enterica from the other two species. Likewise, analysis of the Lane-BC region improves resolution between *Vibrio cholerae *and *V. parahaemolyticus *as well as between *Yersinia pestis *and *Y. entericolitica*. On the other hand, *Brucella *and *Burkholderia *species are better separated by the Lane-AB sequence region.

**Figure 5:**
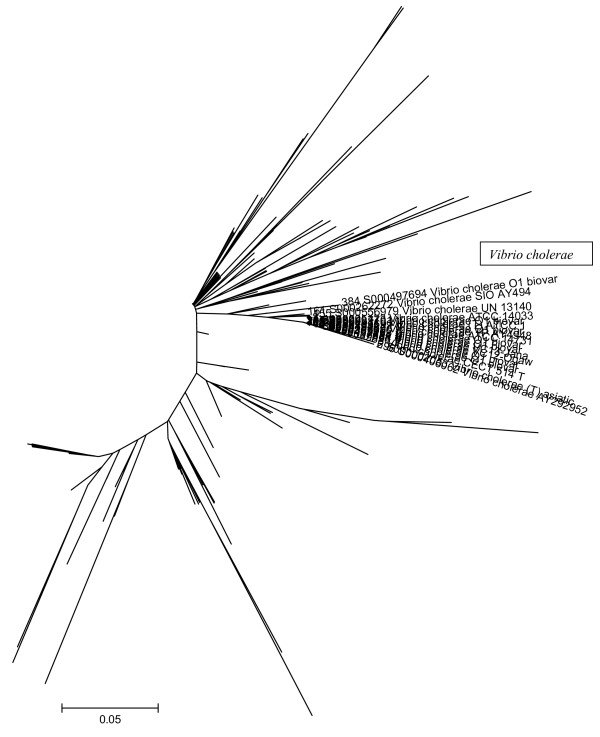
**Demonstration of successful clustering of the pathogen *Vibrio cholerae *among 477 members of the genus *Vibrio *by mass spectrometric observables. **The presented Neighbor-joining (NJ) tree is based on mass-spectral coincidence analysis of the Lane-AB 16S rDNA sequence region of the genus *Vibrio*. Only the names of 20 strains of the pathogen *V.cholerae *are displayed out of 477 amplifiable *Vibrio *species or strains were used for generation of the NJ tree. The mass spectrometric observable distances from near-neighbors presented may be used to determine the limit of discrimination for a given pathogen and base-specific cleavage reaction.

**Figure 6:**
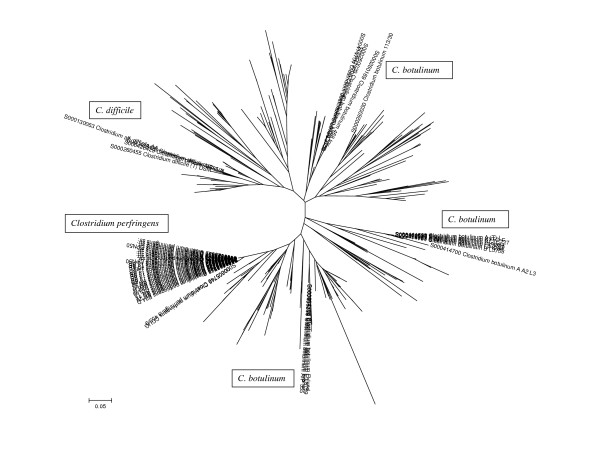
**Neighbor-joining tree based on coincidence analysis of the Lane-AB sequence region of the genus *Clostridium *(549 total species or strains). **For clarity, only *C. botulinum, C. difficile, and C. perfringens *are labeled.

To investigate more closely the extent to which the Category ABC pathogens might be resolved from their innocuous relatives using just a single base-specific cleavage reaction, we used the methods described above to analyze entire genera including pathogens of interest and their close relatives. Figure 5 illustrates that 20 *V. cholerae *strains can be distinguished based on an observable Lane-AB RNase T_1 _fragmentation pattern in the context of 457 other "universally" amplifiable *Vibrio *strains. For clarity, only *V. cholerae*strains are labeled by strain name in Figure 5. As can be seen, none of the 20 *V. cholerae *strains examined were found in branches containing any of the other 457 *Vibrio *species or strains under consideration. Figure 6 depicts a similar analysis of genus *Clostridium *in which three pathogens of interest in this genus are labeled: *C. botulinum, C. difficile*, and *C. perfringens*. Figure 6 shows the "Lane-AB/RNase T_1_" mass fragment tree and the distribution of the three pathogens of interest. While *C. difficile*, and *C. perfringens *cluster nicely relative to other strains, *C. botulinum *is distributed throughout the tree indicating that base-specific mass fragment analysis of the Lane-AB amplicon may be ineffective in discriminating this organism from other *Clostridia*.

## Discussion

### Occurrence of universal primers

Since we did not account for sequences which may have contained one or more indeterminate bases within the primer substring, it is likely that the published primer sequences occur in more strains than reported here. For the purposes of a broad bacterial identification platform, in practice, the primers will likely be employed under conditions of "permissive" PCR in which up to several mismatches will still result in a product, extending the organism coverage of a primer pair by reduced stringency. In any event, many other universally conserved sequences have been proposed for phylogenetic positioning of bacteria [41] and the methods described here are certainly amenable to various primer pairs. Indeed, for some applications it may be best to design custom primer pairs to achieve the best possible resolution between target species.

### Over-represented organisms

To some extent the standard deviations in coincidence (as well as the averages themselves) presented in Figures 1 and 2 may be affected by the prevalence of certain "popular" strains or species in the RDP such as *E. coli *(represented by 80 accessions of length 1200 nt or longer). Conversely, just as sequence-variability may be over-estimated due to a shortage of information on a particular genus, so might the variation in mass-spectral coincidence. Although the coincidences at the genus-level for *Campylobacter *and *Clostridium *stand out as lower than the rest (see Table 2) no attempt was made to normalize the presented coincidences by the number of times a species was used in a calculation. Finally, the lower values of coincidence for the *C.botulinum *lineage, even at the species-level, helps to explain the difficulty in distinguishing that organism from its near-neighbors.

### Masses employed and isotopic distribution

Determination of RNA compositions is challenging because of the small mass difference between U and C. (The repeating G, A, U, and C monomer masses for RNA are respectively: 345.2, 329.2, 306.2, and 305.2 with pair wise differences 16, 39, 40, 23, 24, and 1 Da). In preliminary experimental work, we have routinely incorporated amino-allyl uridine residues as a 100% substitute for natural U in RNA transcripts thereby increasing the 1 Da difference between U and C to 55 Da. Under 100% amino-allyl U (aaU) modification all the fragments are separated by at least ~8 Da. This 8 Da difference is not attributable to a single monomer difference in composition but rather occurs when a C and one aaU residue in one fragment are replaced by two As in a second fragment of otherwise identical composition, e.g. fragment 1, AA*UU*CG = 2048.4 Da, and fragment 2, *CUUU*CG = 2056.4 Da. Thus, incorporation of amino-allyl uridine will provide a significant increase in the resolving power of the mass spectrometric approach.

Those familiar with mass spectrometry of large biomolecules will also appreciate that isotopic distribution of the molecular masses to be measured is an important consideration. We and others have previously published detailed discussions of these issues [31,42]. Experimentally, we have found that isotopic distributions do not prevent accurate organism identification, and use of average masses gives very acceptable results. Finally, automated spectral processing algorithms for "centroiding" vastly reduce the complexity of observed spectra by placing an idealized peak with a peak-width of zero at the center-of-mass of each major peak in the spectrum. We have therefore used average monomer masses in calculating all fragment masses, and amino-allyl U in place of natural U. While the spectral coincidence values calculated here would be the same regardless of the mass used for U, experimentally, as discussed above, the use of amino-allyl U in place of natural U facilitates resolution of fragments with only a single U/C difference in composition (and their underlying isotopic distributions).

In all cases, we assumed the RNase T_1 _digests to be complete. That is, no fragments containing an internal G-residue remained. In practice, significant incomplete digestion due to secondary structure of RNA would be readily recognizable because there would be many more large fragments than expected. Such incomplete digestion was never a problem in the original rRNA digest cataloging studies [21-25] and heavy masses that would be symptomatic of incomplete digestion have not been encountered in our experimental studies.

Although approximately 1% of bases in the naturally occurring 16S rRNA are known to be post-transcriptionally modified and therefore have unexpected masses, the possible presence of such modified nucleotides was not considered here, for several reasons. First, it is known that very few modifications occur in the larger RNase fragments (length 9 or more) [43]. Second, the fragments that contain the modifications and the modification itself are typically highly conserved, and it would therefore be straightforward to identify them and make the needed mass adjustment. Finally, because *in vitro *transcription is likely to be used to generate sufficient 16S rRNA to meet the sensitivity limits of contemporary MALDI instruments, the actual samples will not contain the biological modifications.

Using these methods then, we feel the trees presented are reasonable representations of the theoretical limit of resolution of the method when all expected masses are observed and no unexpected masses are observed, and the nearest two masses in any observed spectrum are well resolved.

### Use of coincidence function with real spectra

The above discussion points out perhaps the most useful aspect of the coincidence function and trees presented here. The inner-product-based metric can be used to compare peak lists from real spectra to large predicted databases of fragments (and we have now routinely used this to rapidly identify bacteria in less than 40 minutes beyond the initial PCR step). Von Wintzingerode *et al*. described comparison of base-specific fragmentation patterns derived from *Bordetella *species against the patterns predicted by virtual fragmentation of 50 published 16S rDNA sequences, including 13 sequences which were known to be closely related [27]. Discriminating masses (those non-degenerate between the strains under consideration) were compared and strains were typed by inspection. Lefmann, et al. used similar methods to rank the identification of mycobacteria [30]. Using the coincidence function described herein with the following tolerance parameter

δ(k)={1|k|≤tol0otherwise     (2b)
 MathType@MTEF@5@5@+=feaafiart1ev1aaatCvAUfKttLearuWrP9MDH5MBPbIqV92AaeXatLxBI9gBaebbnrfifHhDYfgasaacH8akY=wiFfYdH8Gipec8Eeeu0xXdbba9frFj0=OqFfea0dXdd9vqai=hGuQ8kuc9pgc9s8qqaq=dirpe0xb9q8qiLsFr0=vr0=vr0dc8meaabaqaciaacaGaaeqabaqabeGadaaakeaaiiGacqWF0oazcqGGOaakcqWGRbWAcqGGPaqkcqGH9aqpdaGabaqaauaabeqaciaaaeaacqaIXaqmaeaacqGG8baFcqWGRbWAcqGG8baFcqGHKjYOcqWG0baDcqWGVbWBcqWGSbaBaeaacqaIWaamaeaacqWGVbWBcqWG0baDcqWGObaAcqWGLbqzcqWGYbGCcqWG3bWDcqWGPbqAcqWGZbWCcqWGLbqzaaaacaGL7baacaWLjaGaaCzcamaabmaabaGaeGOmaiJaeeOyaigacaGLOaGaayzkaaaaaa@5153@

we have successfully identified bacteria in the laboratory by comparison of acquired fragment patterns to mass catalogs corresponding to over 47,000 rDNA sequences in a rapid, automated, and quantitative fashion. In practice, for mass calibration, an internal fragment common to all reactions is generated from the reverse compliment of the antisense primer. (This mass also serves as a confirmation that the RNA transcription was full-length). In general, such a single point calibration results in sufficient mass accuracy that when the *tol *parameter is set to 1.0 Da or less, correct organism identification is still obtained.

By repeated acquisition of spectra from known model organisms, it should be possible to relate the fidelity of real spectra (that is the number of expected and unexpected peaks observed) to the branch lengths calculated by distance-based clustering methods such as neighbor-joining. Low fidelity spectra will obviously limit organism-resolution, and branches beyond a certain threshold distance would effectively be collapsed. Finally, the results of such cluster analyses should help us to develop faster reporting of organism identification when comparing observed spectra to predicted mass catalogs. Comparison of observed mass spectra to subsamples of mass catalogs from smaller, *mass-based *organism clusters of the database will speed our identification routines.

## Conclusion

Cluster analysis based on mass spectrometric observables is an extremely useful tool for examining the extent to which specificity is preserved in rapid, mass-based assays in comparison with results obtained using complete sequences. The analysis also facilitates evaluation of the relative utility of alternative choices of the region to be amplified. In contrast to, for example, pair wise lists of organisms which have matching or closely matching mass spectra, the trees presented here provide a quick means for visually assessing the resolution achieved using a particular sequence region and cleavage after a particular base. These trees indicate that complete cleavage after just one base using primer pairs that produce 20–30 informative fragments should provide at least genus-level resolution for most bacteria. This resolution can be readily improved by analysis of a second 16S rRNA sequence region, transcription and fragmentation of the antisense strand, and/or cleavage after an alternative base. (A number of methods have been described for generating RNA transcripts which are mass-modified and RNA may be cleaved mono-specifically after bases other than G [26,28,44]). For the purposes of developing a broad-based "sentinel" bacterial assay, this level of resolution may be acceptable, especially if a result is provided rapidly. In situations, for example, in which an enriched unknown substance is presented, whether the rapid analysis indicates *Bacillus anthracis, B. cereus*, or several other near phylogenetic neighbors may be irrelevant to the near-term prophylactic steps to be taken if an assay is sufficiently rapid. In the case of clinical diagnostics and response, many antibiotics have broad organism activity, so a rapid, genus-level diagnostic test will often be of higher resolution than actually needed. Finally, the results of such cluster analyses should help us to develop faster reporting of organism identification by serving as a template for database segmentation when comparing observed spectra to large numbers of predicted mass catalogs.

## Methods

All 16S rDNA sequences used in this study were downloaded in FASTA format from the Ribosomal Database Project, release 9.32. All downloaded sequences were 1200 nt or longer with all alignment gaps removed. For many initial sequence manipulations of FASTA files, the freely available program BioEdit was used [45]. Multiple sequence alignments were performed using the CLUSTALW algorithm [46] within BioEdit using the default gap opening and gap extension penalties. The number of pair wise distances for n organisms is n(n-1)/2. For groups containing large numbers of organisms, such as the phylum Firmicutes, with over 20,000 sequences in the RDP, this would lead to over 200 million pair wise distances. Average coincidences were therefore calculated by taking random samples of 200 sequences. For many other groups of sequences, such as the explicitly named Category A, B, or C pathogens, only several hundred sequences were typically involved. In these cases, the entire sequence set was used for coincidence analysis or tree generation. For the generation of Figure 2, following multiple sequence alignments, positional entropies were calculated as described within BioEdit [39,40] using default parameters. Briefly, entropy in BioEdit gives a measure of uncertainty at each position relative to other positions and is calculated as *H(l) *= -∑*f(b, l)*ln(*f(b, l)*) where *f(b, l) *is the frequency at which residue *b *is found at position *l*. The resulting entropy values at each position in the alignment of a particular phylogenetic grouping of sequences were then averaged as a single measure of the variability of the sequence region in the group.

The freely available program, Molecular Evolutionary Genetics Analysis or "MEGA" v3.1 [38] was used to generate all neighbor-joining (NJ) trees presented. NJ trees were generated using the default parameters and the MEGA 3.1 implementation of the NJ algorithm [32,47].

A suite of custom Linux shell scripts, C programs, and Matlab/Octave functions were written under Linux 2.2.13 and used to create and analyze mass fragment databases. Complete or partial unaligned sequences were downloaded in FASTA format and split into separate files with unique identifier tags which were cross referenced in an index. Each sequence file consisted of a single string composed of A's, G's, C's, and/or T's and was searched for the existence of two (forward and reverse) primer strings. For sequences which contained both strings, new strings representing the inter-primer regions (including the primer sequences themselves) were placed along with the relative position of the first primer into separate files which were also cataloged by the unique identifiers. Next, these "amplicons" were subject to base-specific cleavage after every G residue, and the resulting sequence fragments were used to compute a list of masses corresponding to those fragments. Finally, based on previous determination of the minimum informative length for a given fragment [31], the mass lists were "high-pass" filtered for fragments which were 6-mers or longer, and the resultant lists were cataloged in separate files using their unique identifier tags.

In order to perform quantitative "distance" analysis based on mass-fragment spectra, the mass lists generated above were concatenated into a single file whose first column contained integers representing the origin of the masses and whose second column contained the corresponding lists of masses. Octave (version 2.0.17) could then be used to read this file and generate the square matrix representing the coincidence (using an implementation of the coincidence function introduced previously) between each pair of mass spectra in the file. The resulting matrix was manipulated into a vector which contained the corresponding distances (distance ≡ 1 – coincidence) and which conformed to the structure required for the MEGA 3.1 input file. Additional Linux shell scripts then created a MEGA-compatible file by generating the appropriate header information, relating the unique identifiers back to organism names, and attaching the distance output list.

From start to finish, the entire process can be accomplished automatically by specifying the name of a FASTA formatted file containing sequence data, the desired forward and reverse primers for amplification, and the desired nucleotide(s) for cleavage. The amplification process includes the ability to accommodate arbitrary degrees of primer degeneracy (for example by specifying an 'N' in one of the primer strings), and the mass calculation can be tailored to include mass modified nucleotides, for example, aminoallyl-U for uridine. For an input file containing 220 bacterial 16S sequences (~2,000 bp each), the entire process took just under 3 minutes on an older 1 GHz AMD Athlon (32-bit) personal computer with 512 MB of RAM. The same analysis of 403 complete viral genomes (~10,000 bp each) took approximately 6 minutes.

## Authors' contributions

GWJ conceived of generating trees based on mass-spectral coincidence, carried out an initial analysis based on smaller sets of sequences, created the initial draft manuscript, and revised the manuscript prior to its submission. RJM introduced the coincidence function and inner product, performed all computer programming, and helped revise the manuscript. GEF consulted on the conventional alignments, reviewed the novel trees presented here, and helped draft the manuscript. RCW conceived the overall study, and participated in its design and coordination and helped to draft the manuscript. All authors read and approved the final manuscript.

## Figures and Tables

**Table 1:** Coverage and amplicon characteristics for the adjacent Weisburg and Lane universal primer sets in 47,257 bacterial sequences.

**Forward Primer**	**Reverse Primer**	**Occurrence of Primer Pair in All Bacterial Sequences**	**Occurrence of Primer Pair in Enteric Sequences**	**Average Amplicon Length and Std. Dev. (All Bacteria)**	**Average Amplicon Length and Std. Dev. (Enterics only)**
"Weisburg FWD" AGAGTTTGATCCTGGCTCAG AGAGTTTGATCATGGCTCAG AGAGTTTGATCCTGGCTTAG AGAATTTGATCTTGGTTCAG	"Lane A" CAGCAGCCGCGGTAATAC CAGCAGCCGCGGTAATTC CAGCCGCCGCGGTAATAC CAGCCGCCGCGGTAATTC	9337/47257 = 19.76%	333/1655 = 20.12%	518.78 ± 37.94 bp	530.42 ± 3.38 bp
"Lane A" CAGCAGCCGCGGTAATAC CAGCAGCCGCGGTAATTC CAGCCGCCGCGGTAATAC CAGCCGCCGCGGTAATTC	"Lane B" AAACTCAAAGGAATTGACGG AAACTCAAATGAATTGACGG AAACTTAAAGGAATTGACGG AAACTTAAATGAATTGACGG	38310/47257 = 81.07%	1553/1655 = 93.84%	408.83 ± 7.35 bp	409.17 ± 2.40 bp
"Lane B" AAACTCAAAGGAATTGACGG AAACTCAAATGAATTGACGG AAACTTAAAGGAATTGACGG AAACTTAAATGAATTGACGG	"Lane C" GTACACACCGCCCGT GCACACACCGCCCGT	36,058/47,257 = 76.30%	1401/1655 = 84.65%	500.83 ± 13.94 bp	501.26 ± 1.06 bp
"Lane C" GTACACACCGCCCGT GCACACACCGCCCGT	"Weisburg REV" GGTTGGATCACCTCCTTA AAGTCGTAACAAGGTAACCGT AAGTCGTAACAAGGTAGCCGT AAGTCGTAACAAGGTATCCGT	9991/42257 = 21.14%	373/1655 = 22.54%	123.62 ± 57.24 bp	122.24 ± 8.1978 bp

**Table 2:** Average mass spectral coincidence values for 11 representative organisms at the genus and species levels.

	**Average Coincidence**	
	
**Organism**	species	genus
*Bacillus anthracis*	0.999	0.988
*Bacillus cereus*	0.990	

*Campylobacter jejuni*	0.979	0.512

*Clostridium botulinum*	0.719	0.610
*Clostridium difficile*	0.875	
*Clostridium perfringens*	0.996	

*Escherichia coli*	0.992	0.990

*Salmonella enterica*	0.964	0.955
*Salmonella typhimurium*	0.993	

*Staphylococcus aureus*	0.994	0.939
*Vibrio cholerae*	0.981	0.909

## References

[B1] Ghoshal U, Prasad KN, Singh M, Tiwari DP, Ayyagari A (2004). A comparative evaluation of phenotypic and molecular methods for the detection of oxacillin resistance in coagulase-negative staphylococci. J Infect Chemother.

[B2] Ellepola AN, Hurst SF, Elie CM, Morrison CJ (2003). Rapid and unequivocal differentiation of Candida dubliniensis from other Candida species using species-specific DNA probes: comparison with phenotypic identification methods. Oral Microbiol Immunol.

[B3] Willis RC (2004). Improved molecular techniques help researchers diagnose microbial conditions.. Modern Drug Discovery.

[B4] Cannone JJ, Subramanian S, Schnare MN, Collett JR, D'Souza LM, Du Y, Feng B, Lin N, Madabusi LV, Muller KM, Pande N, Shang Z, Yu N, Gutell RR (2002). The Comparative RNA Web (CRW) Site: An Online Database of Comparative Sequence and Structure Information for Ribosomal, Intron, and other RNAs. BioMed Central Bioinformatics.

[B5] Cole JR, Chai B, Farris RJ, Wang Q, Kulam SA, McGarrell DM, Garrity GM, Tiedje JM (2005). The Ribosomal Database Project (RDP-II): sequences and tools for high-throughput rRNA analysis. Nucleic Acids Res.

[B6] Amann R, Fuchs BM, Behrens S (2001). The identification of microorganisms by fluorescence in situ hybridization. Curr Opin Biotechnol.

[B7] DeLong EF, Wickham GS, Pace NR (1989). Phylogenetic stains: ribosomal RNA-based probes for the identification of single microbial cells. Science.

[B8] Wagner M, Horn M, Daims H (2003). Fluorescence in situ hybridisation for the identification and characterisation of prokaryotes. Current Opinion in Microbiology.

[B9] Busti E, Bordoni R, Castiglioni B, Monciardini P, Sosio M, Donadio S, Consolandi C, Rossi Bernardi L, Battaglia C, De Bellis G (2002). Bacterial discrimination by means of a universal array approach mediated by LDR (ligase detection reaction). BMC Microbiol.

[B10] Uchida T, Bonen L, Schaup HW, Lewis BJ, Zablen L, Woese C (1974). The use of ribonuclease U2 in RNA sequence determination. Some corrections in the catalog of oligomers produced by ribonuclease T1 digestion of Escherichia coli 16S ribosomal RNA. J Mol Evol.

[B11] Chandler DP, Newton GJ, Small JA, Daly DS (2003). Sequence versus Structure for the Direct Detection of 16S rRNA on Planar Oligonucleotide Microarrays. Appl Environ Microbiol.

[B12] Applied Biosystems I MicroSeq 16S rRNA Gene Kit..

[B13] Applied Biosystems I MicroSeq Full Gene 16S rDNA Bacterial Identification Kit..

[B14] Kwon Y, Tang K, Cantor C, Koster H, Kang C (2001). DNA sequencing and genotyping by transcriptional synthesis of chain-terminated RNA ladders and MALDI-TOF mass spectrometry. Nucleic Acids Res.

[B15] Roskey MT, Juhasz P, Smirnov IP, Takach EJ, Martin SA, Haff LA (1996). DNA sequencing by delayed extraction-matrix-assisted laser desorption/ionization time of flight mass spectrometry. Proc Natl Acad Sci U S A.

[B16] Spottke B, Gross J, Galla HJ, Hillenkamp F (2004). Reverse Sanger sequencing of RNA by MALDI-TOF mass spectrometry after solid phase purification. Nucleic Acids Res.

[B17] Koster (1997). DNA diagnostic (sic) based on mass spectrometry.. United States patent 5,605,798 and continuations.

[B18] Koster (2000). DNA diagnostics based on mass spectrometry.. United States patent 6,043,031 and continuations.

[B19] Ecker DJ, Sampath R, Blyn LB, Eshoo MW, Ivy C, Ecker JA, Libby B, Samant V, Sannes-Lowery KA, Melton RE, Russell K, Freed N, Barrozo C, Wu J, Rudnick K, Desai A, Moradi E, Knize DJ, Robbins DW, Hannis JC, Harrell PM, Massire C, Hall TA, Jiang Y, Ranken R, Drader JJ, White N, McNeil JA, Crooke ST, Hofstadler SA (2005). Rapid identification and strain-typing of respiratory pathogens for epidemic surveillance. Proc Natl Acad Sci U S A.

[B20] Hofstadler SA, Sampath R, Blyn LB, Eshoo MW, Hall TA, Jiang Y, Drader JJ, Hannis JC, Sannes-Lowery KA, Cummins LL (2005). TIGER: the universal biosensor. International Journal of Mass Spectrometry.

[B21] Fox GE, Pechman KR, Woese CR (1977). Comparative cataloguing of 16S ribosomal RNA: molecular approach to prokaryotic systematics.. International Journal of Systemic Bacteriology.

[B22] Fox GE, Stackebrandt E, Hespell RB, Gibson J, Maniloff J, Dyer TA, Wolfe RS, Balch WE, Tanner RS, Magrum LJ, Zablen LB, Blakemore R, Gupta R, Bonen L, Lewis BJ, Stahl DA, Luehrsen KR, Chen KN, Woese CR (1980). The phylogeny of prokaryotes. Science.

[B23] Woese CR, Fox GE (1977). Phylogenetic structure of the prokaryotic domain: the primary kingdoms. Proc Natl Acad Sci U S A.

[B24] Woese CR, Fox GE, Zablen L, Uchida T, Bonen L, Pechman K, Lewis BJ, Stahl D (1975). Conservation of primary structure in 16S ribosomal RNA. Nature.

[B25] Woese CR, Stackebrandt E, Macke TJ, Fox GE (1985). A phylogenetic definition of the major eubacterial taxa. Syst Appl Microbiol.

[B26] Hahner S, Ludemann HC, Kirpekar F, Nordhoff E, Roepstorff P, Galla HJ, Hillenkamp F (1997). Matrix-assisted laser desorption/ionization mass spectrometry (MALDI) of endonuclease digests of RNA. Nucleic Acids Res.

[B27] von Wintzingerode F, Bocker S, Schlotelburg C, Chiu NH, Storm N, Jurinke C, Cantor CR, Gobel UB, van den Boom D (2002). Base-specific fragmentation of amplified 16S rRNA genes analyzed by mass spectrometry: a tool for rapid bacterial identification. Proc Natl Acad Sci U S A.

[B28] Krebs S, Medugorac I, Seichter D, Forster M (2003). RNaseCut: a MALDI mass spectrometry-based method for SNP discovery. Nucleic Acids Res.

[B29] Hartmer R, Storm N, Boecker S, Rodi CP, Hillenkamp F, Jurinke C, van den Boom D (2003). RNase T1 mediated base-specific cleavage and MALDI-TOF MS for high-throughput comparative sequence analysis. Nucleic Acids Res.

[B30] Lefmann M, Honisch C, Bocker S, Storm N, von Wintzingerode F, Schlotelburg C, Moter A, van den Boom D, Gobel UB (2004). Novel mass spectrometry-based tool for genotypic identification of mycobacteria. J Clin Microbiol.

[B31] Zhang Z, Jackson GW, Fox GE, Willson RC (2006). Microbial Identification by Mass Cataloging. BMC Bioinformatics.

[B32] Saitou N, Nei M (1987). The neighbor-joining method:  A new method for reconstructing phylogenetic trees.. Molecular Biology and Evolution.

[B33] Weisburg WG, Barns SM, Pelletier DA, Lane DJ (1991). 16S ribosomal DNA amplification for phylogenetic study. J Bacteriol.

[B34] Lane DJ, Pace B, Olsen GJ, Stahl DA, Sogin ML, Pace NR (1985). Rapid determination of 16S ribosomal RNA sequences for phylogenetic analyses. Proc Natl Acad Sci U S A.

[B35] NIAID Category A, B, & C Priority Pathogens. http://www3.niaid.nih.gov/biodefense/bandc_priority.htm.

[B36] NIAID biodefense research agenda for CDC category A agents.. http://www3.niaid.nih.gov/biodefense/research/biotresearchagenda.pdf.

[B37] (2002). NIAID strategic plan for biodefense research.. NIH publication No 03-5306.

[B38] Kumar S, Tamura K, Nei M (2004). MEGA3: Integrated software for Molecular Evolutionary Genetics Analysis and sequence alignment. Briefings in Bioinformatics.

[B39] Pierce JR (1980). An Introduction to Information Theory: Symbols, Signals and Noise.

[B40] Schneider TD, Stephens RM (1990). Sequence Logos: A new Way to Display Consensus Sequences. Nucleic Acids Res.

[B41] Santos SR, Ochman H (2004). Identification and phylogenetic sorting of bacterial lineages with universally conserved genes and proteins.. Environmental Microbiology.

[B42] Koomen JM, Russell WK, Tichy SE, Russell DH (2002). Accurate mass measurement of DNA oligonucleotide ions using high-resolution time-of-flight mass spectrometry. J Mass Spectrom.

[B43] Rozenski J, Crain PF, McCloskey JA (1999). The RNA Modification Database: 1999 update. Nucleic Acids Res.

[B44] Stanssens P, Zabeau M, Meersseman G, Remes G, Gansemans Y, Storm N, Hartmer R, Honisch C, Rodi CP, Bocker S, van den Boom D (2004). High-throughput MALDI-TOF discovery of genomic sequence polymorphisms. Genome Res.

[B45] Hall TA (1999). BioEdit: a user-friendly biological sequence alignment editor and analysis program for Windows 95/98/NT.. Nucl Acids Symp Ser.

[B46] Thompson JD, Higgins DG, Gibson TJ (1994). CLUSTAL W: improving the sensitivity of progressive multiple sequence alignment through sequence weighting, position-specific gap penalties and weight matrix choice. Nucleic Acids Res.

[B47] Nei M, Kumar S (2000). Molecular Evolution and Phylogenetics.

